# Nano-selenium, organic selenium, and novel selenium sources: Which is best for clean and sustainable poultry production?

**DOI:** 10.1016/j.psj.2026.107785

**Published:** 2026-09-16

**Authors:** Ahmed Mohamed Fouad, HebatAllah Kasem El-Senousey, Xin Li, Haobin Hou, Xiaoliang Wang, Yang Lu, Junfeng Yao

**Affiliations:** a Institute of Animal Husbandry and Veterinary Medicine, Shanghai Academy of Agricultural Sciences, Shanghai, 201106, China; b Department of Animal Production, Faculty of Agriculture, Cairo University, Giza, 12613, Egypt

**Keywords:** Selenium sources, Egg quality, Meat quality, Stressors, Sustainability

## Abstract

Selenium (Se) is one of the most widely used feed additives in poultry farms because Se is required to activate antioxidant enzymes, specifically glutathione peroxidase and various types of proteins called selenoproteins. Thus, Se plays several roles such as maintaining the efficient functioning of poultry organs by protecting their cell membranes against oxidative damage caused by free radicals and other chemical compounds generated during normal metabolism. Se supplementation is commonly applied to boost fertility in poultry breeders’ farms. Se is added to poultry diets to maintain poultry productivity by relieving the negative impacts of undesirable conditions such as heat stress, high stocking density stress, vaccination, mycotoxins, and heavy metal contamination. Moreover, Se is required by the human body and the daily Se requirement can be readily satisfied by consuming cheap products such as poultry meat and eggs, so poultry producers must supplement poultry diets with Se to produce suitable meat and eggs for humans. Different Se sources are available on the market and several attempts have also been made to produce novel Se sources in laboratories. These diverse sources cause confusion among poultry producers when selecting a Se source. Therefore, it is necessary to identify Se sources that are the best choices to allow poultry producers to achieve different aims without deleterious impacts on the environment. Consequently, the aims of this review are to help poultry producers to select suitable Se sources, support companies in the production of Se and provide initial evaluations of Se products to enable poultry producers to immediately obtain information from the label, and aid researchers in the development of novel competitive Se sources.

## Introduction

In poultry nutrition, selenium (Se) mostly affects the functioning of body organs, including the brain, cerebellum, thalamus, heart, lung, liver, kidney, pancreas, spleen, thymus, bursa of Fabricius, intestine, testes, ovary, and muscles (Baowei et al., 2011; Zhang et al., 2019; Liu et al., 2022; Du et al., 2024; Haseeb et al., 2025). The concentration of Se is the lowest compared with other trace minerals in poultry diets, but Se-deficient diets lead to significant declines in the Se levels in avian species, decreasing the activities of antioxidant enzymes, including glutathione peroxidase (GSH-Px), catalase (CAT), and superoxide dismutase (SOD). Consequently, significant increases occur in the production of reactive oxygen species and lipid peroxidation, as well as alterations in cytokine production to promote inflammation, apoptosis, and DNA damage in immune organs, such as the bursa of Fabricius, spleen, and thymus (Zhang et al., 2012; Sun et al., 2024; Xu et al., 2024: Wang et al., 2025).

Birds respond to reduced dietary Se by lowering the production of antioxidant enzymes in the trachea and lung, and significant reductions in the Se levels in these organs suppress the synthesis of antibodies and increase the levels of reactive oxygen species, thereby leading to lipid peroxidation, inflammation, and apoptosis (Du et al., 2024; Peng et al., 2023; Yin et al., 2023). Lesions have been detected in the duodenum, jejunum, and ileum in birds fed Se-deficient diets due to inflammation and apoptosis, as well as significant reductions in antimicrobial peptide and tight junction proteins, and disordered antioxidant status (Gao et al., 2016; Liu et al., 2022; He et al., 2023; Yujiao et al., 2023; Luo et al., 2024). In addition, injuries are found in the liver, pancreas, and heart due to oxidative stress and inflammation induced by feeding birds low Se diets (Shi et al., 2023; Ji et al., 2024; Sun et al., 2025). Moreover, decreased Se contents, oxidative damage, and apoptosis are found in the brain, ovary, and testes (Xu et al., 2013; Huang et al., 2016; Liu et al., 2023, 2023), and thus the levels of growth hormone, insulin-like growth factor (Wang et al., 2018), and reproductive hormones, including testosterone (Li et al., 2020), follicle-stimulating hormone (FSH), luteinizing hormone (LH), estradiol, and progesterone, are reduced in birds fed Se-deficient diets (Qin et al., 2026). Thus, the provision of inadequate Se concentrations in poultry diets inhibits the growth rate (Yang et al., 2023), egg production (Liu et al., 2020; Muhammad et al., 2021; Qin et al., 2026), and fertility (Fouad et al., 2020; Manafi et al., 2024), and impairs the meat color and water-holding capacity because of the elevated permeability of cell membranes in breast muscle and myoglobin oxidation (Baowei et al., 2011; Chen et al., 2026) due to disordered glutathione redox balance, mitochondrial functions, and ATP production, and accumulation of excessive reactive oxygen species (Wu et al., 2023; Liu et al., 2025), with lipid and protein oxidation in muscles (Wang et al., 2009; Mohamed et al., 2020).

Se is present in most of the commercial cereal grains and beans used for formulating poultry diets, but its concentration is low and insufficient to satisfy the needs of poultry (NRC, 1994). Poultry premix contains Se in an inorganic form, which is insufficient for biological functions due to its low bioavailability (Surai et al., 2018). Thus, poultry producers must supplement Se from high bioavailability sources to maintain productivity and reproduction in poultry under commercial conditions including stressors, and in order to satisfy the specific demand of customers for meat and eggs enriched with Se (Seremelis et al., 2019; Michalczuk et al., 2021; Lima et al., 2025; Wen et al., 2026). Se-enriched yeast, Se-methionine, selenocysteine, hydroxy‑selenomethionine, 2‑hydroxy-4-methylselenobutanoic acid, and nano-Se are commercially available, and novel Se sources are produced in laboratories such as Se-enriched probiotic (Pan et al., 2011; Zhou et al., 2025), Se-enriched earthworm hydrolysate (Cheng et al., 2025), Se-rich black soldier fly (Zhang et al., 2024), Se-enriched yellow mealworms powder (Qiu et al., 2021), Se-chitosan (Lari et al., 2024), selenized glucose (Mohammadsadeghi et al., 2024), Se-enriched *Cardamine violifolia* (Wang et al., 2023), Se polysaccharides (Bo et al., 2021; Liu et al., 2023; Wang et al., 2025; Lin et al., 2026), and other novel Se sources (Luo et al., 2025; Shahidin et al., 2025). These sources of Se can confuse poultry producers when selecting an appropriate Se source. Therefore, the main aims of this review are to help poultry producers select appropriate Se sources, as well as allowing companies and researchers to make suitable Se products, and to provide useful information about their products based on measurements in poultry, as shown in Figure 1.

**Fig. 1:**
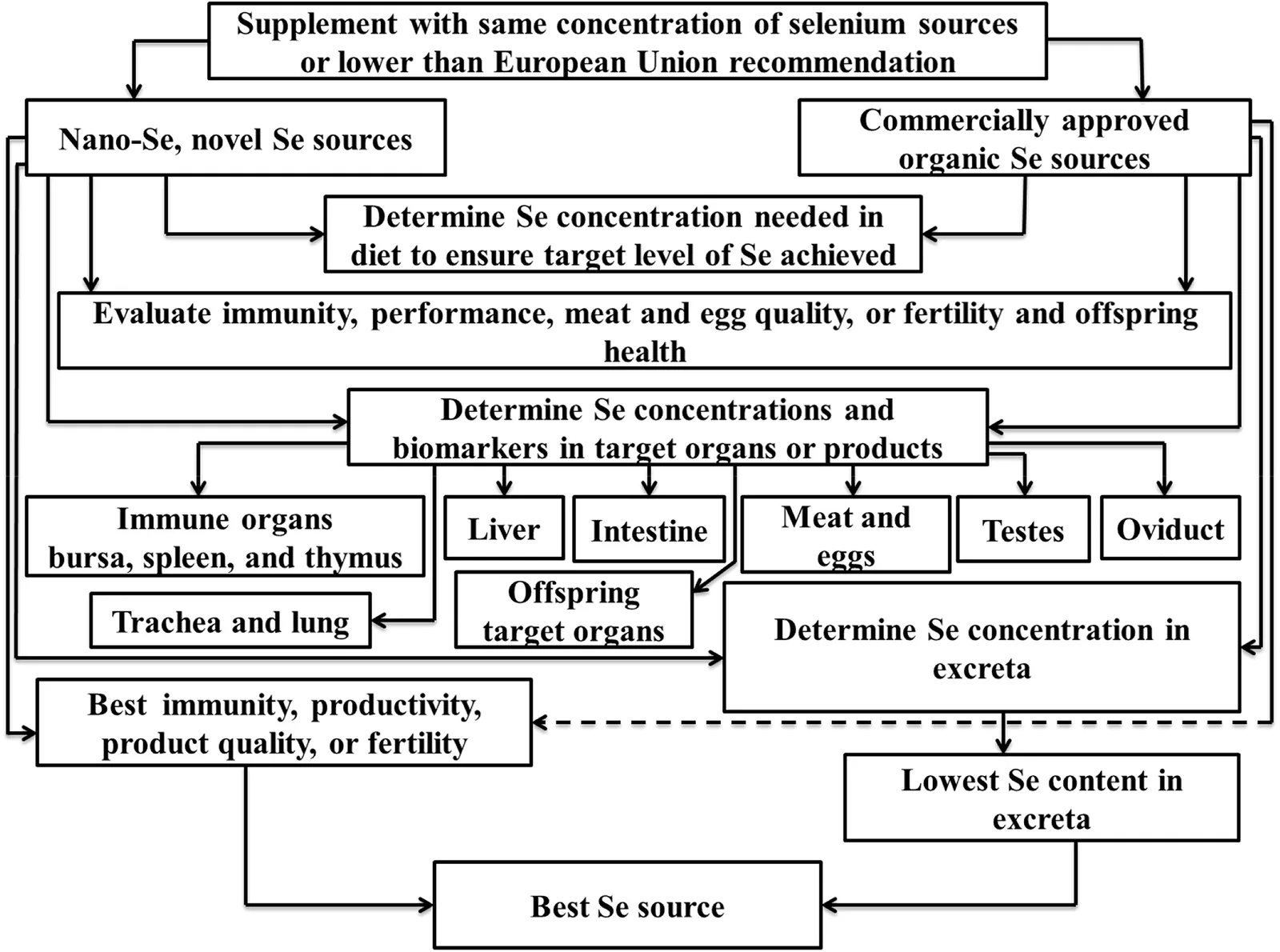
Identify the best Selenium source.

### Se sources and egg yield

As shown in Table 1, adding 0.3 up to 10.0 mg Se/kg from organic, inorganic Se sources, nano-Se, and novel organic Se sources, including Se-enriched lactobacilli, Se-enriched *Lactobacillus plantarum*, Se-enriched yellow mealworms powder, Se-rich black soldier fly, and Se-enriched earthworm hydrolysate, did not affect the egg yield compared with the control, but supplementation with 0.3, 0.4, or 0.5 mg Se/kg from traditional organic sources, inorganic sources, nano-Se, or new organic sources, such as Se-enriched polysaccharides from *Phellinus linteus*, Se-enriched *Cardamine violifolia*, and selenized glucose, enhanced the feed conversion ratio (Meng et al., 2021; Wang et al., 2023) and egg yield (Liu et al., 2020; Muhammad et al., 2021, 2022; Liu et al., 2023; Mohammadsadeghi et al., 2024; Li et al., 2025; Qin et al., 2026). Supplementing Se in basal diets deficient in Se can significantly modulate the productive performance. Adding an appropriate level of Se to the diets of laying hens was shown to increase the deposition of Se in the intestine, liver, and different parts of the reproductive system (Liu et al., 2022, 2023;Li et al., 2025; Qin et al., 2026). Se supplementation also led to improvements in the antioxidant status of these organs, increases in beneficial intestinal bacteria, such as *Lactobacillus, Veillonella*, and *Turicibacter*, and decreases in the abundances of deleterious bacteria such as *Stenotrophomonas* (Liu et al., 2022), as well as maintaining the normal secretion of reproductive hormones and upregulating their receptors in the ovary (Qin et al., 2026). These effects may explain why Se influences the egg yield and why different Se sources have different impacts on the egg yield.

**Table 1:** Selenium sources and productivity of egg-type birds.

Among the new organic Se sources, supplementation with Se-chitosan in the diet of laying birds at a level of 0.6 mg/kg improved the egg yield, egg weight, and feed conversion ratio (Lari et al., 2024; Mohammadsadeghi et al., 2023). The Se levels that improved productivity in studies by Lari et al. (2024) and Mohammadsadeghi et al. (2023) are higher than that recommended for poultry by the European union. One of the most important properties of the Se source employed in the latter studies was its highly bioavailability compared with other sources at the same concentration, although this was not specifically tested in these studies. High levels of Se can be added to poultry diets to enhance their productivity, but the Se concentrations in excreta should be measured and possible environmental pollution with Se needs to be considered.

Among the various available Se sources, only supplementation with Se-enriched yeast improved the egg yield, egg weight, and feed conversion ratio in a study by Chen et al. (2024), whereas Li et al. (2025) found that Se-enriched yeast, Se-methionine, and nano-Se had similar effects on boosting egg production compared with the control. In the following sections, we consider why the application of commercial organic Se sources in egg production does not obtain identical results in terms of performance, as well as meat quality in meat-type birds.

### Se sources and egg quality

As shown in Table 2, studies have demonstrated that among external and internal measures of egg quality, the Haugh unit score, yolk color, eggshell strength, egg Se content, and yolk malondialdehyde (MDA) content can be altered by changing the Se source used in the diets of laying hens. The Haugh unit score, yolk color, and yolk MDA content are evaluated to assess egg freshness and the yolk lipid oxidation status (Tian et al., 2023; Yang et al., 2025), and the egg Se content is linked with the production of functional foods (Wen et al., 2026). An appropriate dietary Se level and Se source can improve the Haugh unit score, yolk color, egg Se content, and eggshell strength, and decrease the yolk MDA content, possibly due to Se boosting the egg antioxidant status, leading to suppressed lipid peroxidation and enhancement of the Haugh unit core and yolk color, and reducing the yolk MDA content (Pan et al., 2011; Lari et al., 2024; Mohammadsadeghi et al., 2023; 2024).

**Table 2:** Impacts of selenium sources on egg quality.

Supplementation with a suitable dietary Se level and source can enhance intestinal populations of specific beneficial bacteria, such as *Lactobacillus, Bifidobacteria, Veillonella*, and *Turicibacter* and decrease those of specific deleterious bacteria, such as *Escherichia coli, Salmonella, Stenotrophomonas*, and *Enterococcus* (Liu et al., 2022; Lari et al., 2024; Muhammad et al., 2021; Mohammadsadeghi et al., 2023; 2024). These changes could positively reflect the bioavailability of nutrients required for eggshell formation and explain why Se enhances the eggshell quality.

Previous studies have demonstrated that organic Se is more effective than inorganic Se in improving egg quality economic traits. The egg Se content is one of the most important egg quality economic traits and other egg quality measurements are not always affected when organic Se sources are changed but the egg Se content is influenced by altering the bioavailability of Se in the diet. For example, Pan et al. (2011) showed that supplementation with 0.5 mg Se-enriched probiotics/kg resulted in lower egg Se contents compared with adding the same level of Se using l-Se-methionine or Se-enriched yeast as the source (Delezie et al., 2014). The basal diet used in the study by Delezie et al. (2014) contained a higher Se concentration than that employed by Pan et al. (2011), but increasing the Se concentration to 1.0 mg/kg led to the production of eggs containing 582 μg Se/kg according to Pan et al. (2011) and 615 μg Se/kg when supplemented with Se at only 0.5 mg/kg according to Delezie et al. (2014). Thus, l-Se-methionine was more efficient at producing eggs enriched with Se. The yolk color was not significantly affected by supplementation with Se-enriched yeast or Se-methionine, but the egg Se content was significantly increased by supplementation with Se-methionine (Słupczyńska et al., 2018). Irrespective of the breed and experimental period, the egg Se contents were higher in hens fed diets supplemented with Se-methionine or Se-yeast (Słupczyńska et al., 2018) compared with hens fed diets supplemented with Se-enriched yeast (Han et al., 2017). The egg Se content was not significantly affected by supplementing hen’s diets with Se-enriched yeast or nano-Se (Meng et al., 2021), but Liu et al. (2020) found that adding Se-enriched yeast led to the deposition of almost double the Se content in eggs compared with addition of Se-enriched yeast in the study by Meng et al. (2021). The egg quality and yolk Se content were similar after dietary supplementation of Se-enriched yeast or Se-enriched probiotic (Muhammad et al., 2021), whereas supplemental Se organic sources such as glycine nano-Se or Se-enriched *Cardamine violifolia* did not affect the egg quality compared with inorganic Se (Zhou et al., 2021; Wang et al., 2023). Shu et al. (2026) showed that including various levels of Se in the form of nano-Se chitosan to hen’s diets failed to affect the egg quality and egg Se content compared with diets supplemented with inorganic Se. In addition, Yan et al. (2025) found that the yolk Se content was significantly lower when hens consumed diets supplemented with Se-enriched *Cardamine violifolia* compared with hens that consumed diets supplemented with inorganic Se. By contrast, supplementing the diets of laying birds with organic Se sources, such as selenized glucose, Se chitosan, or Se-rich black soldier fly, enhanced the egg Se content compared with using inorganic Se (Lari et al., 2024; Mohammadsadeghi et al., 2023; Mohammadsadeghi et al., 2024; Zhang et al., 2024). Thus, it is necessary to determine which new Se organic sources perform better, such as selenized glucose, Se chitosan, and Se-rich black soldier fly, compared with commercial organic sources, such as Se-enriched yeast and Se-methionine. Supplementation with Se-enriched yeast increased the egg Se content (Qin et al., 2026) but including Se-enriched yeast at the same level in the diet of hens increased the yolk and egg Se contents by more than 2.5–3 times (Li et al., 2024; Yan et al., 2025; Wang et al., 2025) compared with the results reported by Qin et al. (2026). The Se-enriched yeast product employed by Qin et al. (2026) contained the highest Se concentration compared with the Se-enriched yeast products supplemented by Yan et al. (2025) and Wang et al. (2025). These findings indicate that Se absorption, utilization, and its impacts do not depend only on the Se concentration in organic product, but instead the organic component such as the yeast strain, environmental production conditions, including the materials and production steps, purity of the final product, and concentrations of Se-methionine and other Se-compounds in the final product may affect the quality and impact of the organic Se source. Indeed, Chen et al. (2024) compared products comprising Se-yeast types A, C, and N, Se-methionine, and nano-Se, which contained 4955, 2295, 2707, 2000, and 5321 mg Se/kg, respectively. They demonstrated that hens fed diets supplemented with Se-methionine containing only 2000 mg Se/kg produced eggs that had the highest Se content compared with other Se sources. The egg Se content was highest when hens were fed diets containing Se-methionine compared with diets containing Se-cysteine, nano-Se, and inorganic Se (Chen et al., 2025). Moreover, the egg Se content was highest in hens fed diets supplemented with Se-enriched yeast compared with diets supplemented with Se-methionine and nano-Se (Li et al., 2025). The Se concentrations were identical at 20000 mg/kg in the Se-cysteine and Se-methionine products (Chen et al., 2025), whereas the Se concentration in the nano-Se product was 30000 mg/kg. These results show that only considering the Se concentration in a product is insufficient for evaluating Se sources.

The forms of amino acids (L-amino acid vs. DL-amino acid) can affect the efficiency and subsequently bioavailability of Se. In particular, it was shown that l-methionine was more efficient at improving the economic traits of poultry compared with DL-methionine (Zhang et al., 2019; Teng et al., 2023), but the forms of Se-methionine and Se-cysteine used in some previous studies were not stated. Wu et al. (2011) found that the egg Se content was significantly higher when breeder hens were fed diets supplemented with DL-Se-methionine compared with breeder hens fed diets supplemented with l-Se-methionine at the same supplementation level. Zorzetto et al. (2021) showed that only adding 0.2 mg/kg hydroxy‑Se-methionine to the diets of breeder hens obtained the same egg Se content as including 0.3 mg/kg inorganic Se in their diet. Therefore, adding Se-methionine forms with known purity may be effective, but determining the characteristics of Se-enriched yeast and nano-Se with a known nano-particle size range, percentage, and production method could help identify a source that is better for poultry nutrition. Thus, the nano-Se production method (biological, chemical, or physical) (Ferroudj et al., 2026) and nano-Se particle size range (Feng et al., 2025) must be considered to evaluate nano-Se sources. However, Li et al. (2025) did not state the Se concentration or nano-Se particle size range in the products used in their experiment. Qiu et al. (2021) found that the Se content of eggs produced by hens supplemented with 2.0 mg Se/kg from Se-enriched yellow mealworms powder contained a high Se concentration (4480 mg/kg) and it was higher than that of eggs produced by hens supplemented with 3.0 mg Se/kg from Se-enriched lactobacilli (Zhou et al., 2025). Qiu et al. (2021) and Zhou et al. (2025) supplemented diets with Se levels that were higher four to six times the EU recommendation, but only supplementing Se-enriched yeast at a concentration of 1.5 mg/kg in the diets of hens led to the production of eggs containing a higher Se concentration compared with adding Se-enriched lactobacilli and Se-enriched yellow mealworms powder (Zhou et al., 2025). The chicken breed and experimental period were the same in the studies by Qiu et al. (2021) and Zhou et al. (2025), but adding Se-enriched yeast at only 1.5 mg/kg was better for producing Se-enriched eggs compared with high Se supplementation levels of 6.0 and 10.0 mg/kg in Se-enriched lactobacilli and Se-enriched yellow mealworms powder, respectively. These results show that the absorption, utilization, and impacts of Se are not dependent only on the Se concentration in organic products, but the organic component also has a major role in the effect obtained. Therefore, the selection of a Se supplementation level and Se organic source should not be based only on economic traits, such as the productive performance, egg quality, egg Se content, and Se price, but instead the ecosystem balance and sustainability should be considered by determining the Se contents of excreta.

### Se sources and meat yield and quality

As shown in Table 3, in most studies, the nano-Se supplementation level recommended for local breeds and commercial broilers was 0.3 mg/kg, but Ibrahim et al. (2019) recommended using 0.6 mg/kg. Therefore, the recommended level of nano-Se in meat-type birds is similar to that recommended for other Se sources. Thus, it is necessary to understand the advantage of supplementing the diets of meat-type birds with nano-Se, which is possible by comparing nano-Se with organic Se sources. Li et al. (2018) and Chen et al. (2024) found that the meat color, Se content of breast, and breast meat juiciness were better when supplementing using organic Se sources such as Se-yeast and Se-methionine compared with nano-Se, whereas the best final body weight gain, feed conversion ratio, and muscle Se content were recorded in broilers fed diets supplemented with 0.6 mg/kg Se-methionine or nano-Se (Ibrahim et al., 2019). Broiler diet supplementation with nano-Se or Se-enriched yeast led to similar significant enhancements in the breast meat juiciness and Se content compared other Se sources (Bakhshalinejad et al., 2019). According to studies by Ferroudj et al. (2025, 2026), under a Se-deficient basal diet, the Se content of breast meat was lower than that in the control, and supplementing with a high level of 5.0 mg/kg nano-Se measuring 80–120 nm in the diet significantly increased the Se content of breast meat (197 μg/kg). Thus, the size of nano-Se particles can affect its bioavailability and performance, and it was shown that the breast meat juiciness and Se content (130 μg/kg) were significantly boosted by supplementation using nano-Se with an average size of 80 nm at 0.4 mg/kg (Elkhateeb et al., 2024). Similarly, Ibrahim et al. (2019) found that supplementation using nano-Se with an average size of 42 nm at a concentration of 0.6 mg/kg improved the body weight gain, breast meat juiciness, and Se content (770 μg/kg). By contrast, the use of nano-organic Se such as Se*-Capsicum annuum* did not affect the performance of broilers and the Se content of meat (Al-Khalaifah et al., 2025). However, supplementation using nano-Se as Se-*Bacillus subtilus* DA20 at 0.04 mg/kg with a particle size of 56 nm improved the final body weight, feed conversion ratio, and carcass yield (Al-Quwaie, 2023). Improvements were obtained by supplementation using nano-Se in the study by Al-Quwaie (2023), but the Se concentrations were not examined in the basal diet, serum, edible part, and meat, or other Se biomarkers. Studies have demonstrated that nano-Se does not always have greater effects on the meat quality and Se content of breast meat than inorganic Se, which could be due to the size of the nano-Se particles and/or the production method. However, organic Se sources such as Se-yeast or Se-methionine obtain equivalent or greater effects on the meat quality and Se content of breast meat compared with nano-Se. For example, Bakhshalinejad et al. (2019) reported that Se-yeast containing 22.4% Se was better than nano-Se containing 32.8% Se for improving the meat juiciness and Se content. Se-yeast, Se-methionine, and 2‑hydroxy-4-methylselenobutanoic acid are the most common organic Se sources, but studies have still not fully compared the effects of new organic Se sources and organic Se. Mohamed et al. (2020) demonstrated that the breast meat with the highest Se content (95 μg/kg) was achieved by supplementing with Se-enriched *Enterobacter cloacae*, and supplementing with high levels of Se-enriched *Bacillus subtilis* and *Bacillus licheniformis* led to the deposition of 230 μg Se/kg in breast meat (Yang et al., 2019). In addition, using other new organic Se sources such as Se-enriched *Candida utilis* was better than Se-enriched yeast for boosting the Se content of breast meat (300 μg/kg) (Wang et al., 2020). Adding Se-enriched *Cardamine violifolia* did not affect the performance of broilers or the meat quality compared with Se-yeast (Xu et al., 2022), and Se-enriched kale sprouts led to the accumulation of a lower Se content (1030 μg/kg) in breast meat compared with Se-methionine (Chantiratikul et al., 2021). Therefore, it is necessary to understand why new organic Se sources such as Se-enriched *Candida utilis* perform better than Se-enriched yeast in enhancing the Se content of meat, and to assess studies that compared the effects of Se-enriched yeast and Se-methionine on the Se content of meat to identify the best organic Se source for meat-type birds.

**Table 3:** Impacts of selenium sources on performance and meat quality (Bami et al., 2022; Bień et al., 2023; Cai et al., 2012; Cheng et al., 2026; Fan et al., 2026; Lu et al., 2020; Wang et al., 2022; Zhang et al., 2020; Zhou and Wang, 2011; Wang et al., 2022).

Briens et al. (2013, 2014), Couloigner et al. (2015), and Zhao et al. (2017) found that 2‑hydroxy-4-methylselenobutanoic acid was significantly better than Se-enriched yeast for enhancing Se deposition in meat. It would be useful to know whether the Se-enriched yeast employed in these studies and other studies had similar compositions in order to recommend 2‑hydroxy-4-methylselenobutanoic acid rather than Se-enriched yeast. De Marco et al. (2021) showed that supplementation with the same concentrations of l-Se-methionine, 2‑hydroxy-4-methylselenobutanoic acid, and two-types of Se-enriched yeast containing different concentrations of Se-methionine in broiler diets led to the deposition of significantly more Se in breast meat compared with the Se-enriched yeast source that contained the highest Se-methionine concentration. In addition, Liu et al. (2025) showed that among several Se sources, supplementing Se-methionine led to the highest Se content in breast meat. De Marco et al. (2021) found that the Se-methionine concentration in Se-enriched yeast sources affected the bioavailability of Se because Se-enriched yeast B contained a high Se-methionine concentration (72%) and significantly more Se was deposited in breast meat compared with that when using Se-enriched yeast A containing only 56% Se-methionine, which may explain the different impacts of Se-enriched yeast supplementation in poultry. Studies have shown that l-Se-methionine and 2‑hydroxy-4-methylselenobutanoic are the organic Se sources with the highest bioavailability, but it is unclear whether they lead to the lowest environmental contamination. Estimating the Se concentration in excreta produced by birds fed diets supplemented with these two organic Se sources could answer this question. The characteristics of Se-enriched yeast products, such as the yeast strain, yeast size, Se concentration, and Se-methionine percentage, as well as the concentrations of other Se-compounds should be defined on the product. Moreover, novel organic Se sources should be compared with other organic Se sources such as l-Se-methionine and 2‑hydroxy-4-methylselenobutanoic to assess their possible advantages. The Se content of excreta needs to be considered when selecting Se sources in order to ensure sustainable poultry production.

### Se sources and fertility

As shown in Table 4. Se-enriched yeast and nano-Se significantly improved the sperm motility and viability compared with inorganic Se, but nano-Se performed best at decreasing lipid peroxidation and improving the total antioxidant capacity (Asghari-Moghadam and Mehri, 2025). By contrast, supplementing with the same concentration of Se from various sources, such as Na-selenite, Se-enriched yeast, and nano-Se, did not significantly affect reproductive hormones, the Se contents of testis, antioxidant status, and sperm density (Yan et al., 2024). Brennan et al. (2012) showed that supplementing Se-yeast led to an increase in the Se concentration in testes compared with Se-deficient basal diet. The semen quality and fertility were not altered by supplementing using the same level of Na-selenite and different sources of Se-yeast (Sabzian-Melei et al., 2022), whereas the semen quality and fertility percentage were boosted (Manafi et al., 2024), and the activities of antioxidant enzymes were enhanced and lipid peroxidation reduced in the testes of roosters fed diets supplemented with hydroxy‑Se-methionine and Se-methionine, respectively (Long et al., 2022). Thus, it is necessary to clarify the best organic Se source for enhancing the fertility of roosters. Ashraf et al. (2020) found no significant differences in using Se-yeast, hydroxy‑Se-methionine, and Se-methionine for improving the semen quality characteristics, but these organic sources performed significantly better than inorganic Se. The main target when improving the antioxidant status of testes and decreasing lipid peroxidation is boosting the semen quality to maximize fertility (Fouad et al., 2020), but most studies did not examine the percentage fertility. The new organic Se source comprising Se-enriched *Ganoderma lucidum* was tested in roosters (Chen et al., 2026) but they did not record the percentage fertility and recommended using a high level of this Se source without determining the Se concentrations in excreta. Improvements in the sperm motility, viability, and antioxidant status, and reduced lipid peroxidation are not inevitable consequences of boosted fertility. Li et al. (2025) demonstrated that supplementing Se-yeast significantly enhanced the testosterone level, number of Sertoli cells, antioxidant status, and semen quality, such as the sperm moving angle and sperm viability, as well as suppressing lipid peroxidation, but the percentage fertility was not affected. Therefore, Se sources should be compared to identify the best for enhancing fertility in male poultry by evaluating the semen quality and percentage fertility, and estimating the Se concentration in excreta.

**Table 4:** Impacts of selenium sources on fertility.

### Se sources and stressors

As shown in Table 5, organic Se sources including Se-methionine, Se-enriched yeast, and OH-Se-methionine improved the productive performance and egg quality metrics, such as the Haugh unit score and egg shell thickness, in heat-stressed egg-type birds. The use of organic Se in stressed hens could have reduced the production of inflammatory cytokines and corticosterone secretion, and boosted the antioxidant defense system, leading to decreased lipid peroxidation and apoptosis, and helping maintain intestinal, liver, and oviduct functions (Abbas et al., 2022; Gao et al., 2026; Yang et al., 2025). Supplementation with Se in heat-stressed birds activated Se deposition in the eggs and promoted the synthesis of alkaline phosphatase, as well as elevating the blood concentrations of calcium and phosphorus (de Brito et al., 2023; Ferit Gursu et al., 2003; Sahin et al., 2008). These changes could explain why supplementation with organic Se led to improvements in the Haugh unit score and eggshell thickness in birds exposed to heat stress. By contrast, nano-Se did not affect the productive performance and egg quality, but inorganic Se reduced the percentage of broken eggs (Chen et al., 2025). In heat-stressed meat-type birds, supplementing with nano-Se at 1.2 mg/kg improved the feed conversion ratio (Safdari-Rostamabad et al., 2017), while adding nano-Se alginate oligosaccharides at 5 mg/kg reduced the breast frozen loss (Yang et al., 2023), and supplementing nano-Se at 0.2–0.3 mg/kg was sufficient to improve the performance, antioxidant capacity, and breast Se content (Mahmoud et al., 2016; Lochi et al., 2023, 2024). In addition, supplementing nano-Se with particle sizes ranging from 40 to 75 nm at a concentration of 0.15 mg/kg led to similar enhancements in the final body weight and feed conversion ratio as adding the same concentration of an inorganic Se source (Rehman et al., 2022). Thus, it is necessary to determine the most suitable size for nano-Se particles and the best organic compounds for use in producing nano-Se with high bioavailability in poultry. Nano-Se with a particle size of 60 nm and a source containing a higher Se concentration compared with organic Se sources, but supplementing with nano-Se resulted in the equivalent liver Se content (Wang et al., 2023) and a significantly lower breast Se content compared with supplementing using organic Se at the same concentration (Jing et al., 2024). Supplementing organic Se sources including Se-enriched yeast, Se-enriched probiotics, Se-methionine, and OH-Se-methionine at levels ranging from 0.15 to 0.3 mg/kg improved the productivity (Sun et al., 2021; Chen et al., 2022) and meat quality in stressed meat-type birds (Liao et al., 2012; Mahmoud et al., 2016; Khan et al., 2018).

**Table 5:** Impacts of selenium sources on stressed poultry.

Similar to the supplementation levels used for organic Se sources, adding 0.3 mg/kg of a new organic Se source comprising Se-enriched *Enteromorpha* polysaccharide mitigated the oxidative stress induced by lipopolysaccharide and enhanced the performance of broilers (Li et al., 2026). However, alleviating the immunosuppression induced by cyclophosphamide and enhancing the body weight gain, feed conversion ratio, production of antibodies, and relative weights of the immune organs required supplementation with Se-*Broussonetia papyrifera* polysaccharide at 1 g/kg (Xu et al., 2025). Similar findings were obtained in vaccine-stressed birds where the oral administration of Se-lily polysaccharides, Se-*Brassica rapa* L. polysaccharide, or Se-garlic polysaccharides boosted the Newcastle disease antibody titer, lymphocyte development, and levels of inflammatory cytokines, such as IL-2, IL6, and INF-γ (Hou et al., 2016; Bo et al., 2021; Wang et al., 2025). Similarly, Lin et al. (2026) showed that the oral administration of Se-*Hericium erinaceus* polysaccharides enhanced the immune organs and intestinal development, synthesis of antibodies and inflammatory cytokines, and Newcastle disease antibody levels in birds vaccinated against Newcastle disease (Lin et al., 2026). These laboratory-produced new organic Se sources were effective in experiments, but they still need to be fully compared with commercially approved organic Se sources. Rao et al. (2013) showed that adding an organic Se source to the diets of vaccinated chickens significantly improved the proliferation of lymphocytes, whereas adding Se-enriched yeast to the diets of chickens vaccinated against avian influenza resulted in the highest production of antibodies against avian influenza compared with that using inorganic Se (Shojadoost et al., 2020).

Supplementing commercial nano-Se at a concentration of 0.5 mg/kg significantly increased the egg yield and egg weight, and improved the feed conversion ratio and egg quality in hens fed diets contaminated with mercury (Ma et al., 2022ab), possibly because nano-Se inhibited the accumulation of this heavy metal in prehierarchical follicles, thereby decreasing apoptosis by follicular granulosa cells and promoting the secretion of reproductive hormones, including progesterone, estradiol, and follicle-stimulating hormone (Cheng et al., 2023; Ma et al., 2022b). In addition, supplementing 0.2 mg/kg nano-Se decreased oxidative stress induced in the ovary and enhanced the secretion of reproductive hormones in quails that consumed water contaminated with lead (Eleouny et al., 2026). Nano-Se significantly decreased the deposition of mercury in various body organs (Ma et al., 2022a,b), which may have been related to the modulatory effect of Se on the detoxification system, although the impacts of different Se sources on detoxification systems in birds exposed to heavy metal toxicity have not yet been tested. Supplementing 0.2 mg/kg nano-Se with particle sizes of 30–100 nm or inorganic Se improved the daily weight gain and feed conversion ratio in quails that consumed diets contaminated with 1 mg/kg aflatoxins B_1_ (Khazraei et al., 2022), possibly due to the decreased production of the most toxic form of aflatoxin B1 (AFB1-8,9-epoxide) by boosting the antioxidant defense system and detoxification system (Fouad et al., 2019). Moreover, adding nano-Se with average particle sizes of 30–60 nm at a concentration of 1 mg/kg or Se-yeast at a concentration 0.4 mg/kg in broilers injected with ochratoxin-A at 50 μg/kg led to improvements in body weight gain and the feed conversion ratio through suppressing cecal lipid peroxidation and inflammatory cytokine production, and enhancing cecal integrity by upregulating the expression of tight junction proteins (Fang et al., 2023; Yang et al., 2020). However, supplementation with 0.5 mg/kg hydroxy Se-methionine did not affect the body weight or antioxidant status of broilers that consumed diets containing 2 mg/kg ochratoxin-A (Kulcsár et al., 2025), possibly because the experimental period was short and a trial of 5 days was insufficient to affect the productive performance. Thus, the productive performance was not affected by ochratoxin-A compared with the basal diet without ochratoxin-A injection in the study by Kulcsár et al. (2025). Studies have not tested the protective effects of different sizes of nano-Se particles and organic Se sources on poultry that consume feed contaminated with mycotoxins. The recommended supplementation level for nano-Se is higher than or equal to the recommended level for organic Se in stressed poultry. Therefore, it is not clear whether nano-Se is a competitive source with respect to organic Se sources. Thus, detecting Se, aflatoxin B_1_, and ochratoxin-A deposition in the edible parts of poultry and excreta could facilitate evaluations and help select the most suitable size for nano-Se particles and the best organic Se source to mitigate the deleterious impacts of mycotoxins in poultry, as well as the most eco-friendly Se source.

## Conclusion

The characteristics of organic selenium products should be stated on the products, including the production processes and properties of the materials used for production, such as the yeast strain, yeast size, and forms of amino acids (L-amino acid vs. DL-amino acid), as well as the purity of the final product and concentrations of Se, Se-methionine, and other Se-compounds. Similar to organic Se products, nano-Se products should state the production method, nanoparticle size, percentage of each nanoparticle size, purity of the final product, and Se concentration. In future research, it is necessary to identify the most suitable size of nano-Se particle for use in supplements for poultry diets in order to achieve supplementation target outcomes with the minimum concentration compared with organic Se sources. Comparisons are required among novel Se sources and organic Se sources with known characteristics in order to assess their advantages. The most suitable Se source should be characterized by high bioavailability and a low Se content in excreta. Thus, a key challenge is identifying novel Se sources that achieve the same impacts as the best available Se sources, but with the lowest supplement concentration in poultry diets and lowest residual Se content in excreta.

## Authors' contributions

Ahmed Mohamed Fouad was responsible for conceiving and designing the study, conducting a literature search, and reviewing and editing the manuscript. HebatAllah Kasem El-Senousey helped with designing the study, conducting the literature search, extracting the data, interpreting the findings and reviewing the manuscript. Xin Li helped with designing the methodology, the literature search process, interpreting the results and reviewing the manuscript. Haobin Hou helped with investigation, methodology, visualization, original draft preparation. Xiaoliang Wang participated in data curation, investigation, methodology, visualization, and original draft preparation. Yang Lu participated in data curation, visualization, methodology, writing-original draft. Junfeng Yao conceived and supervised this study, contributed to fund acquisition, reviewed and finalized the manuscript. All authors read and approved the final manuscript. All authors agree to be personally and publicly responsible for all aspects of the work.

## Disclosures

All authors declare no conflict of interest.
